# Imaging zebrafish neural circuitry from whole brain to synapse

**DOI:** 10.3389/fncir.2013.00076

**Published:** 2013-04-24

**Authors:** Louis C. Leung, Gordon X. Wang, Philippe Mourrain

**Affiliations:** Department of Psychiatry and Behavioral Sciences, Center for Sleep Sciences, Beckman Center, Stanford UniversityPalo Alto, CA, USA

**Keywords:** zebrafish, clinical neuroscience, psychiatry, calcium imaging, synapse imaging, array tomography, whole brain imaging

## Abstract

Recent advances in imaging tools are inspiring zebrafish researchers to tackle ever more ambitious questions in the neurosciences. Behaviorally fundamental conserved neural networks can now be potentially studied using zebrafish from a brain-wide scale to molecular resolution. In this perspective, we offer a roadmap by which a zebrafish researcher can navigate the course from collecting neural activities across the brain associated with a behavior, to unraveling molecular identities and testing the functional relevance of active neurons. In doing so, important insights will be gained as to how neural networks generate behaviors and assimilate changes in synaptic connectivity.

## INTRODUCTION

One of the major goals of neuroscience is to understand how the nervous system, as a dynamic assembly of cells and connections, generates behaviors as a suite of motor outputs. Impressive progress has been made in recent times in understanding how the nervous system develops and functions. However, with the current set of animal models, neuroscience is approaching a problem of how one can simultaneously work and integrate data across the different scales and modalities at which one can interrogate brain function. To understand a neural process across the scales–from molecules, synapses, neurons, networks to whole brain–is a *bona fide* frontier in the neurosciences today.

**FIGURE 1:**
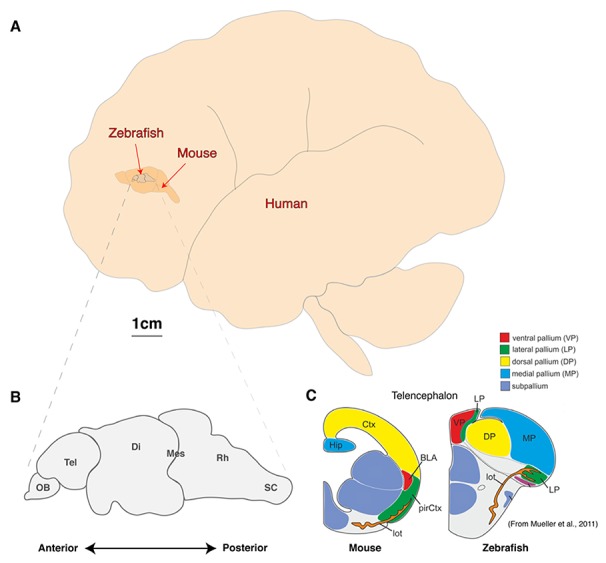
**Comparison of brain sizes of common vertebrate neuroscience models.** **(A)** Schematic diagram of the lateral view of adult human, mouse, and zebrafish brains to scale. **(B, C)** Schematic diagram of the linear organization of major brain regions from olfactory bulb to spinal cord **(B)** and the dorsal location of telencephalic structures such as the presumptive hippocampus and amygdala (C; adapted from Mueller et al., 2011). OB, olfactory bulb; Tel, Telencephalon; Di, Diencephalon; Mes, Mesencephalon; Rh, Rhombencephalon; sc, Spinal cord; Ctx, Neocortex; Hip, Hippocampus, pirCtx, Piriform cortex; BLA, Basolateral Amygdala and lot, lateral olfactory tract.

Advances in functional neuroimaging are allowing us to identify with increasing precision which brain regions are correlated with a particular behavioral output. However, brain-wide visualization, permitted by electroencephalography (EEG) and functional magnetic resonance imaging (fMRI), does not reliably approach the cellular and/or synaptic spatial resolution of brain processing (Thai et al., 2009; Lenkov et al., 2012). Conversely, electrophysiological or high-resolution imaging methods to record neural activity are difficult to extend beyond discrete brain regions. To understand the neural basis of behavior, a challenging goal in basic and clinical neuroscience will be to bridge the gap between these distant levels–i.e., to be able to record and analyze the entire brain with single neuron, if not, single synapse accuracy. Here, we suggest that the recent developments in live whole brain Ca^2^^+^ imaging and super resolution array tomography (AT) can, when applied to a suitably compact brain (**Figure 1**), reveal and correlate whole brain activity maps down to circuit function and changes in the synaptic landscape. Already an established vertebrate model for developmental biology, the zebrafish’s genetic toolbox and unique physical characteristics can now be exploited for the neurosciences.

### THE ZEBRAFISH CAN BE A KEY BRIDGING MODEL FROM CIRCUIT TO MOLECULAR NEUROSCIENCE

As with other animal models, the zebrafish will never fully recapitulate the complex psyche and behaviors of humans. However, the fundamental computational units of brain processing are likely to be conserved and are thus well-studied outside the human brain in model organisms (Sengupta and Samuel, 2009; Bellen et al., 2010; Friedrich et al., 2010; Rinkwitz et al., 2011; Wang et al., 2011).The most common mammalian model used in neuroscience is the mouse, which offers a great variety of complex behaviors, powerful genetics and excellent *ex vivo* brain slice electrophysiological techniques (Kullander, 2005; Van Meer and Raber, 2005; Ward et al., 2011; Kim et al., 2013). However, attempting whole brain modeling with a mouse is a formidable challenge despite being three orders of magnitude smaller than an adult human brain, which has an estimated 10^11^ neurons each making around 10^4^ connections (**Figure 1**). At this time, a genetic model with fewer neurons and a smaller, more accessible brain would be a more feasible option.

**FIGURE 2:**
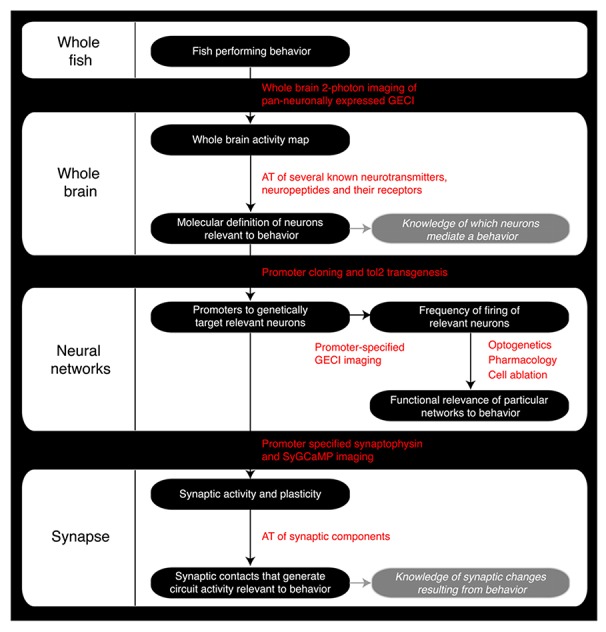
**Roadmap of the steps a zebrafish neuroscience researcher can take to integrate the various levels of analysis within the same model organism.** GECI, Genetically encoded calcium indicator; AT, Array Tomography; tol2, Tol2 transposase; SyGCaMP, Synaptophysin-specific GCaMP sensor.

Zebrafish, sharing conserved neurochemistry and broad brain organization with their mammalian counterparts, may help to bridge this gap and give the first insights of circuit dynamics from whole brain down to molecular changes during conserved behaviors. Five key advantages of studying the zebrafish brain are its (i) compact size, (ii) conservation of the neuropeptide pool (in contrast to invertebrates), (iii) linear organization of brain regions, (iv) structural accessibility of internal nuclei (no overlaying neocortex), and (v) optical clarity (Akanji et al., 1990; Charonis et al., 1990; Appelbaum et al., 2009; Berman et al., 2009; Friedrich et al., 2010; Engert and Wilson, 2012). Particularly with respect to imaging dynamic processes, the zebrafish model uniquely excels as its brain is translucent and small enough that the entire volume can be captured at single cell resolution by standard microscopy magnifications. Even at 6 days old, this represents a formidable 100,000 neurons (Naumann et al., 2010), but the linear organization of the major brain regions from olfactory bulbs to spinal cord tip further facilitates brain-wide imaging (**Figure 1B**). In addition, while the amygdala, hippocampus and habenula are difficult regions to scan in mammals due to their deep location beneath the neocortex, their position is inverted in zebrafish. While the anterior neural tube of mammals undergoes invagination during development, leading to their deep location beneath the neocortex, the eversion process in the development offish telencephalic makes these behaviorally important structures the most dorsal nuclei of the telencephalon (**Figure 1C**; Northcutt, 2008; Mueller et al., 2011). Further, the ease of transgenesis, pharmacological studies, and conservation of behaviors (fear, anxiety, learning and memory, feeding/preying, social and sexual behavior, sleep and diurnality, etc.), altogether makes zebrafish a powerful complement to other models used to study neural processing fundamentals likely to be conserved in humans. As we set out a roadmap below (**Figure 2**), we contend approaches using zebrafish could yield insights to coordinated brain activity and function that underpin the normal healthy brain and what goes awry in pathological contexts.

### WHOLE BRAIN IMAGING OF NEURAL ACTIVITY

The ability to image dynamic cellular and subcellular processes during development has revolutionized the field of developmental biology, ever moving from analyzing fixed samples to dynamic processes in living animals (Huisken and Stainier, 2009; Leung et al., 2011; Randlett et al., 2011; Leung and Holt, 2012). Similarly, the central nervous system is also a highly dynamic entity and adopting methods to interrogate the neural activity of cells and networks at brain-wide, single cell resolution in the context of a behaving animal will be a turning point in systems neuroscience. To characterize the activity landscape of a brain at rest or engaged in a defined behavior on these scales, we suggest the minimum requirements are a neural marker of activity that can be used across the brain without *a priori* hypotheses and an appropriate method to record this activity over time in a specific animal. Following that, an ability to identify these active neurons molecularly is crucial for subsequent progress.

A popular method to mark active neurons involves sacrificing an animal while (or just after) it performs a behavior/process and post-stain for a genetic marker of neural activity-the immediate early genes [IEGs such as cFos and early growth response protein 1 (EGR-1)] – in whole or specific brain regions of interest. This has led to the identification of several nuclei involved in behavioral processes (Hoffman et al., 1993; Sherin et al., 1996; Murphy et al., 2004), but despite good correspondence with neural activity, methods using IEGs have their limitations. Firstly, it is possible that IEGs are only induced when neurons are highly/over activated. Indeed, it is likely that tonic or low firing neurons, which may also be important for a particular computation, may have little or no IEGs induced. Therefore, until further demonstrations of the degree of activation required to induce IEG expression are made, caution should be exercised in concluding that they mark the entire firing population involved in a process. Secondly, a single IEG like cFos is not pan neuronally expressed so it is unlikely to be the sole gene used by *all* the neurons active during a given behavior. Thirdly, there is important computational information encoded when neurons cease firing as opposed to when they become active, and no IEG currently reports the end of a period of activity. Finally, the fixation for IEG staining precludes dynamic information about activity and firing patterns which could provide a crucial handle on the processing and functions carried out by active neurons.

**FIGURE 3:**
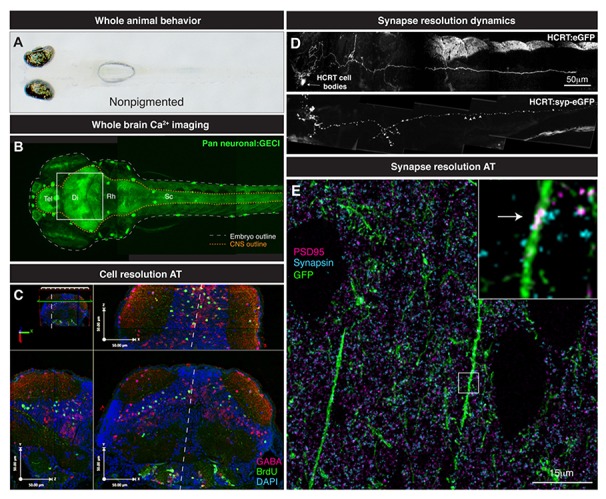
**From whole brain to single synapse.** **(A)** Behavioral processes can be studied in non-pigmented (Nacre; mitfa mutant) species of zebrafish. **(B)** Two-photon volume image of 5 dpf zebrafish expressing GCaMP pan-neuronally, note the linear organization of the brain along the rostrocaudal axis from telencephalon (tel) to spinal cord (sc). **(C)** Various maximal projections (from area indicated in B by white box) of the 5 dpf zebrafish midbrain reconstructed after array tomography against GABA (magenta), BrdU (green) and DAPI (cyan) markers. White dashed line depicts midline. **(D)** Genetically specified circuit analysis. Synaptophysin-eGFP (syp-eGFP) allows imaging of structural plasticity of synapses (white arrowheads) in hypocretin (HCRT) neurons. **(E)** Single synapse resolution projection of array tomography sections stained for GFP (green), PSD95 (magenta) and Synapsin (cyan). Inset, magnification of a single shaft synapse (white arrow).

The development of genetically encoded Ca^2^^+^ indicators (GECIs) sets to solve many of these limitations and zebrafish is particularly suited to the use of GECIs with the ease of genetics/transgenesis and a translucent central nervous system. GECIs, such as GCaMP (Nakai et al., 2001; Muto et al., 2011, 2013) respond with changes in fluorescence intensity proportional to subcellular Ca^2^^+^ changes. In fact, the latest generation of GCaMPs have the ability to reliably indicate single action potential (AP) events and a whole library of versions exist to suit various potential uses (Akerboom et al., 2012; Ohkura et al., 2012) as well as Ca^2^^+^ sensors that operate at UV and red-shifted excitation wavelengths (Zhao et al., 2011; Akerboom et al., 2013). A further advantage of GECIs as compared to traditional electrophysiological recordings is their minimal invasiveness. A multi-electrode array that can discriminate spiking activity from a dozen neurons is prohibitively large for a zebrafish brain, while GECI imaging is completely non-invasive and can acquire spiking activity from hundreds of neurons from one image plane. Combined with pan-neuronal promoters and two-photon excitation microscopy, the monitoring of brain-wide neural activity during brain computations holds great potential (**Figure 3B**). By focusing excitation to small precise volumes in a tissue, two-photon imaging greatly reduces phototoxicity resulting from illumination of tissues above regions of interest typical of epifluorescent and confocal microscopy (Carvalho and Heisenberg, 2009). This permits longer term imaging of living samples than previously possible. An important advantage of operating in the infrared range is that the imaging also does not interfere with light-sensitive behaviors such as circadian and sleep rhythms (Appelbaum et al., 2010). Such excitation also confers benefits to the depth of tissue penetration and thus the imaging of deeper structures in the brain. To give an idea of scale, an entire 1 month juvenile fish brain has a thickness of ~1 mm, which can be fully covered in depth with two-photon scanning. Indeed, the entire brain of a 7 dpf larva can be covered with few imaging frames, as has already been attempted with remarkable success even with the modestly sensitive GCaMP2 (Ahrens et al., 2012). Here, the larva’s computations during changes in motor gain during fictive swimming were located without *a priori* assumptions to specific brain regions such as the inferior olivary complexes. With advanced microscopy, the possibility of whole brain imaging at single cell resolution at physiological frequencies is within reach to zebrafish researchers (Ahrens and Keller, 2013).The recent developments in imaging Ca^2^^+^ sensitive emitters of bioluminescence, such as green fluorescent protein (GFP)-aequorin, in freely behaving zebrafish offers further exciting avenues to complement GCaMP data of stabilized/paralyzed animals (Naumann et al., 2010). After gaining such volumes of information, the upcoming challenge with brain-wide “activity screens” will be to accurately define the neuronal populations of interest. Without accurate knowledge of the neuroanatomical location or access to specified circuits, this remains a challenging hurdle to a true understanding of circuit function.

### IDENTIFYING MOLECULAR MARKERS WITH ARRAY TOMOGRAPHY

So how do we progress from locating the active nuclei correlated with particular behaviors and knowing their molecular identity? What method can provide this missing link? We propose the use of AT to take the same zebrafish that are used in Ca^2^^+^ imaging studies and perform a powerful spatial proteomic approach to gain the molecular identity of the neurons that are active in a given process (**Figure 3C**). AT is an imaging technique that uses nanometer-thin physical sectioning of a fixed tissue sample to facilitate the multiplexed imaging of dozens of protein markers with exquisite spatial resolution and large volumetric tissue coverage (Micheva and Smith, 2007; Wang and Smith, 2012).To date, AT has been broadly used to characterized genetically targeted neurons in zebrafish (Robles et al., 2011), assess the synapse deficits caused by Tau accumulation in mouse and humans (Kopeikina et al., 2011), quantify the three-dimensional microstructural changes of mouse aortic tissue after aneurysm (Saatchi et al., 2012), and measure synapse density changes due to astrocytic glypican release (Allen et al., 2012) and thalamic network stimulation (Lacey et al., 2012).

Conjugated live imaging of neuronal activity and subcellular-level AT will allow the quantitative analysis of cell physiology on a circuit level. AT analysis of cell-type specific antigens will provide single neuron differentiation of cell classes based on transmitter expression, e.g., glutamate, GABA, acetylcholine, etc., (**Figure 3**). Moreover, these transmitter-determined neuron types can be further classified by the expression of neuropeptides or transcription factors. By properly aligning Ca^2^^+^ and AT data, single neuron activity profiles can be mapped from Ca^2^^+^ imaging experiments to molecularly identified neurons. This *post hoc* identification of the molecular physiology of measured circuit members in conjunction with the temporal data from live imaging should allow a more robust classification of functional relevance in terms of understanding how inhibition, excitation and global modulation affect the discrete calculations made by a specific circuit. Further, since the sectioned sample can be kept indefinitely (Micheva and Smith, 2007), when new markers for neuron identity become available, previously unidentified nuclei that displayed interesting activities can be marked and leads to a model that becomes more accurate with time.

The combination of Ca^2^^+^ imaging and AT means we will be able to map functionally relevant brain regions or nuclei in terms of their temporal activity patterns and their molecular topology. When correlated to behavior, the temporal structure could also provide us with a map of how sensory inputs change the active connections of a defined neural circuit. Obtained under both baseline and challenged conditions, firing patterns could also be examined to gain insights to the nature of the disruptions involved in brain disorders and neural degeneration (see Future directions). On top of this functional structure we will then be able to overlay a relevant molecular topology that will reveal the identity of the nodes, whether inhibitory or excitatory, being connected in the functional activity structure. At its simplest, the temporal correlation of inhibitory and excitatory inputs into a circuit will be the basis of the computation performed by that group of neurons, and once both the identity and the activity structures stereotypic to a specific set of inputs and outputs are known, it will then be possible to reconstruct the actual algorithm performed by the circuit in relation to the set of inputs. Moreover, with the wealth of molecular information we can obtain from AT, we will be able to look at how patterns of activity might affect global modulation of the brain through the induction of various categories of neurotransmitters from monoamines to peptides.

Distinguishing the molecular identity of each brain nucleus – if not neuron - is crucial so that we can exploit the use of transgenesis to genetically capture these circuits in order to further study their functions. As with other model organisms, genetic tagging of circuits will be critical since there is significant interindividual variation in the spatial location of neurons that prevents ease of comparison by the activity map alone. With promoter driven transgenic lines at hand, specific neural populations can be imaged in more detail for their firing rates [GCaMP, aequorin and genetically-encoded voltage indicators (GEVIs)] to begin to understand the relative contributions of subgroups or individual neurons to a behavioral output. Further, once relevant firing circuits or nuclei are established, pharmacological treatment, ablations, and optogenetic techniques (for review, Portugues et al., 2013) can be used more accurately to dissect the necessity and sufficiency for those regions (or even single cells) in normal behavioral processes and put us in a position to model brain disorders.

### SYNAPSE IMAGING WITH TWO-PHOTON OR ARRAY TOMOGRAPHY

An ideal understanding of brain computation and function will also require insights at the synaptic level. Perturbations at this level as seen with Schizophrenia, Fragile X syndrome, and Rett syndrome highlight the need to understand what constitutes normal brain connectivity (Chahrour and Zoghbi, 2007; Lauriat and Mcinnes, 2007; Featherstone, 2010; Toro et al., 2010; Auerbach et al., 2011; Grant, 2012). Zebrafish allow a large shift in scale, from whole brain to neural network down to subsynaptic components. For example, zebrafish permit both longitudinal studies of synapses and whole brain coverage of the synaptic landscape. Thanks to the accessibility of the zebrafish brain, live two-photon imaging of genetically specified synaptic populations offers an important glimpse into the dynamics of synapse formation and disassembly related to the function of networks. For example, a longitudinal study of zebrafish hypocretin synapses on axons innervating the pineal gland at larval stages demonstrated rhythmicity of synaptic connections made by this circuit over time (Appelbaum et al., 2010). The reduction in phototoxicity allowed the imaging of the same genetically defined neuronal process in a live vertebrate over 24 h (live zebrafish can be safely imaged in agarose during a full sleep/wake cycle; Appelbaum et al., 2010). Such insights are not possible using fixed samples of several individuals and no other animal model currently offers opportunities to study such phenomenon on a brain-wide scale over time. Ca^2^^+^ dynamics can also be investigated at the synaptic level across the brain or a genetically defined circuit to demonstrate functionality. GCaMP indicators fused to synaptic markers (e.g., SyGCaMP and SyRGECO) have allowed the deciphering of the neural coding involved in the transfer of information between cells in the zebrafish retina (Dreosti et al., 2011) and tectum (Nikolaou et al., 2012; Walker et al., 2013), showing the exciting potential of understanding synaptic firing at specific connections. Such a gain in resolution – access to the firing pattern of individual synapses – bears tremendous potential for revealing the potential disruptions in brain diseases.

As discussed above for circuit dynamics, it is useful to know the molecular identity of an activity profile – and AT again affords this opportunity at the level of the synapse. Such information identifies the nature of these synapses, whether they are excitatory or inhibitory and if they are undergoing plasticity changes such as those for long-term potentiation/long-term depression (LTP/LTD) induction. The sub-diffraction resolution (Wang and Smith, 2012) and the proteomic coverage (Micheva and Smith, 2007) of AT is ideal for the analysis and classification of synapses in a large tissue volume. The combination of live two-photon structural analysis with AT will allow the identification of proteins involved in the structural dynamics of synapses in the brain (**Figure 3D**). A straightforward longitudinal analysis of synapse dynamics (Niell et al., 2004; Appelbaum et al., 2010) followed by *post hoc* AT analysis of the stable or newly formed synapses can give insight into the molecular mechanism by which synapses are stabilized or generated (**Figure 3E**). This further combined with measured activity of those synapses could open the door to finding the molecular and synaptic mechanisms underpinning behavioral control.

In this new regime, perturbations that affect synaptic plasticity, e.g., disease or behavioral challenges, can be characterized during longitudinal analysis, and the affected synapses will be targeted for *post hoc* proteomic dissection to reveal potential molecular changes. Then the candidate molecules can be labeled and modified and put back into a living system and then reanalyzed in terms of their effects on synaptic plasticity and dynamics. In this manner, deep molecular knowledge about the workings and deficits of nervous systems can be gleamed by this iterative process of longitudinal, quantitative observation and conjugate molecular dissection.

## FUTURE DIRECTION AND APPLICATIONS FOR HUMAN DISORDERS

Zebrafish as a genetic model system has driven change in developmental biology and we expect a similar impact in the neurosciences with the advent of whole brain and synapse imaging techniques. While the zebrafish, as with all other animal models, can never accurately recapitulate the behavioral output of a human, we contend that at the level of the synapse and neuron, invaluable insights can be made, with techniques that leverage the unique properties of this vertebrate model, in understanding the basic conserved principles of how neuronal networks coordinate and function.

Knowledge of normal vertebrate brain function will have a huge impact understanding normal brain health and psychiatry. Importantly, there is an increasing realization that some psychiatric/brain diseases are predominantly genetic, developmental and neuronal/synaptic disorders. Indeed, insights to brain health and psychiatry require a whole brain perspective at single synapse resolution. Such a situation may appear to be an unreachable goal today, but zebrafish comes very close to bridging these scales of neural circuit investigation. Psychiatry as a field is now becoming a more integrated field benefiting of neuroscience and genetic studies. The introduction of novel uses of established animal models such as zebrafish in psychiatry and clinical neurosciences should allow new perspective and strategies. With development of psychiatric therapies in decline, advances in our understanding of the molecular basis of synaptic changes in normal and diseased brains should offer new targets for the pharmacological industry. Mental health disorders are the leading cause of disability according to most medical sources. For most mental illnesses, the etiology is unknown, detection and prevention are poor, and current medication is not consistently effective. We hope whole brain studies with synapse resolution in the vertebrate zebrafish will soon allow breakthrough advances in our understanding of complex brain disorders.

## Conflict of Interest Statement

The authors declare that the research was conducted in the absence of any commercial or financial relationships that could be construed as a potential conflict of interest.
